# Inhibition of the Adenosine A_2A_ Receptor Mitigates Excitotoxic Injury in Organotypic Tissue Cultures of the Rat Cochlea

**DOI:** 10.3390/cells8080877

**Published:** 2019-08-12

**Authors:** Belinda RX Han, Shelly CY Lin, Kristan Espinosa, Peter R Thorne, Srdjan M Vlajkovic

**Affiliations:** Department of Physiology and The Eisdell Moore Centre, Faculty of Medical and Health Sciences, The University of Auckland, Private Bag 92019, Auckland 1142, New Zealand

**Keywords:** glutamate excitotoxicity, cochlear synaptopathy, cochlear explant, adenosine A_2A_ receptor, istradefylline, hidden hearing loss

## Abstract

The primary loss of cochlear glutamatergic afferent nerve synapses due to noise or ageing (cochlear neuropathy) often presents as difficulties in speech discrimination in noisy conditions (hidden hearing loss (HHL)). Currently, there is no treatment for this condition. Our previous studies in mice with genetic deletion of the adenosine A_2A_ receptor (A_2A_R) have demonstrated better preservation of cochlear afferent synapses and spiral ganglion neurons after noise exposure compared to wildtype mice. This has informed our current targeted approach to cochlear neuroprotection based on pharmacological inhibition of the A_2A_R. Here, we have used organotypic tissue culture of the Wistar rat cochlea at postnatal day 6 (P6) to model excitotoxic injury induced by *N*-methyl-d-aspartate (NMDA)/kainic acid (NK) treatment for 2 h. The excitotoxic injury was characterised by a reduction in the density of neural processes immediately after NK treatment and loss of afferent synapses in the presence of intact sensory hair cells. The administration of istradefylline (a clinically approved A_2A_R antagonist) reduced deafferentation of inner hair cells and improved the survival of afferent synapses after excitotoxic injury. This study thus provides evidence that A_2A_R inhibition promotes cochlear recovery from excitotoxic injury, and may have implications for the treatment of cochlear neuropathy and prevention of HHL.

## 1. Introduction

Sensorineural hearing loss, for example caused by exposure to noise, is mostly associated with cochlear injury, including the loss of sensory hair cells and primary auditory neurons in the cochlea. The classic view of sensorineural hearing loss is that the hair cell death is the earliest sign of degeneration in both noise- and age-related hearing loss, whilst the degeneration of sensory neurons occurs secondary to the loss of hair cells [1].

More recently, this view has been challenged by evidence that extensive loss of auditory afferent nerve synapses and primary auditory neurons, rather than hair cell death, is an early sign of cochlear damage, and that it can occur as the primary event, even without the loss of sensory hair cells [2,3,4,5,6]. The primary loss of the glutamatergic afferent nerve synapses due to ageing or noise has been named “cochlear neuropathy” or “cochlear synaptopathy” and is thought to occur, at least in part, due to excessive release of glutamate at the inner sensory hair cell—auditory nerve synapse. This form of neural injury does not always lead to a change in auditory thresholds as shown in an audiogram. In animals it manifests as poor detection of sound at higher intensities. In humans it is thought to present as more complex auditory processing deficits such as difficulties in speech discrimination in noisy conditions and may explain declining auditory performance in ageing individuals with normal or near-normal audiograms, now known as “hidden hearing loss” (HHL) [3,5,7]. Degeneration of peripheral auditory fibres also changes the organisation of the ascending auditory pathways resulting in cortical reorganization, which underlies perceptual abnormalities such as tinnitus and hyperacusis [8].

There is currently no effective treatment for HHL. Previous animal studies have attempted to reduce the extent of synaptic damage, and the supplementation of neurotrophic factors (NT-3, Brain-derived neurotrophic factor (BDNF)) was one of the proposed strategies to promote reconnection between hair cells and nerve fibres [9]. Neurotrophin treatment improved the survival of afferent (ribbon) synapses in the cochlea and extended the survival of spiral ganglion neurons [10].

Adenosine receptors and associated signalling pathways are also important mediators of neuroprotection and have been proposed as therapeutic targets in a variety of neurodegenerative diseases [11]. The adenosine receptors (A_1_, A_2A_ and A_3_) are expressed in cochlear tissues [12]. We have previously identified the adenosine A_1_ receptor (A_1_R) as a promising target for the treatment of acute noise-induced cochlear injury [13,14]. Systemic or local administration of A_1_R agonists such as adenosine amine congener (ADAC) and 2-Chloro-N^6^-cyclopentyladenosine (CCPA) in the post-exposure period leads to the improvement of auditory thresholds, reduced expression of oxidative stress markers and increased survival of sensory hair cells in the noise-exposed cochlea [13,14]. A_1_R also seems important in other forms of sensorineural hearing loss such as from cytotoxic drugs, e.g., cisplatin and aminoglycosides [15,16]. This supports our view that A_1_R is an important regulator of cochlear survival in stress and injury [17].

Adenosine A_2A_ receptors (A_2A_R) are also involved in neuroprotection in the central nervous system, predominantly as negative regulators. Studies in mice lacking the A_2A_R (*adora2a*) gene show reduced neuronal injury after occlusion of the middle cerebral artery, and neuroprotection in several models of neurodegenerative diseases [18,19]. Similarly, A_2A_R antagonists confer neuroprotection in animal models of stroke, Parkinson’s disease, Alzheimer’s disease and traumatic brain injury, most likely by controlling neuroinflammation, excitotoxic neuronal damage and synaptopathy [18]. Our recent study on adenosine A_2A_R-null mice has demonstrated better preservation of outer hair cells and afferent synapses in the cochlea and the minimal loss of spiral ganglion neurons after noise exposure compared to wildtype mice [20]. This raises the possibility that the inhibition of A_2A_R could be potential therapeutic target for cochlear neuropathy.

To test this we investigated the effect of A_2A_R inhibition on the development of excitotoxic neural injury using an organotypic tissue culture model of the rat cochlea. Excitotoxic injury was induced by addition of *N*-methyl-d-aspartate (NMDA) and kainic acid in tissue culture medium. A_2A_R were inhibited using istradefylline (KW-6002), the first adenosine A_2A_R antagonist that has been approved clinically for the treatment of l-DOPA-resistant patients with Parkinson’s disease [21,22,23]. This drug has high affinity and selectivity for A_2A_R and excellent safety profile in human studies [11,23]. Here, we report that istradefylline mitigates excitotoxic injury and prevents deafferentation of the inner hair cells in an organotypic tissue culture model.

## 2. Materials and Methods

### 2.1. Animals

Male and female Wistar rats at postnatal day 6 (P6) were used in this study. The animals were obtained from the animal facility at the University of Auckland. All experimental procedures were carried out with the approval of the University of Auckland Animal Ethics Committee, in agreement with the Animal Welfare Act (1999).

### 2.2. Tissue Preparation and Organotypic Tissue Cultures

After decapitation, the cochleae were removed from the temporal bones and dissected in ice-cold sterile 0.1 M phosphate-buffered saline (PBS). The lateral wall, the Reissner’s membrane and the tectorial membrane were removed, and the sensory epithelium was carefully dissected to preserve afferent connections with the spiral ganglion. The cochlear explants were maintained on collagen-coated coverslips in N-2 supplemented Dulbecco’s modified Eagle medium (10% DMEM, Thermo Fisher, Waltham, MA, USA) with the addition of 10% foetal bovine serum (FBS, Gibco Invitrogen, Dublin, Ireland) and 30 units/mL of penicillin (Sigma Aldrich, St. Louis, MO, USA). The cochlear explants were incubated in normal culture medium for 44 h at 37 °C with 5% CO_2_.

### 2.3. Induction of Excitotoxic Injury and Istradefylline Treatment

**Figure 1:**
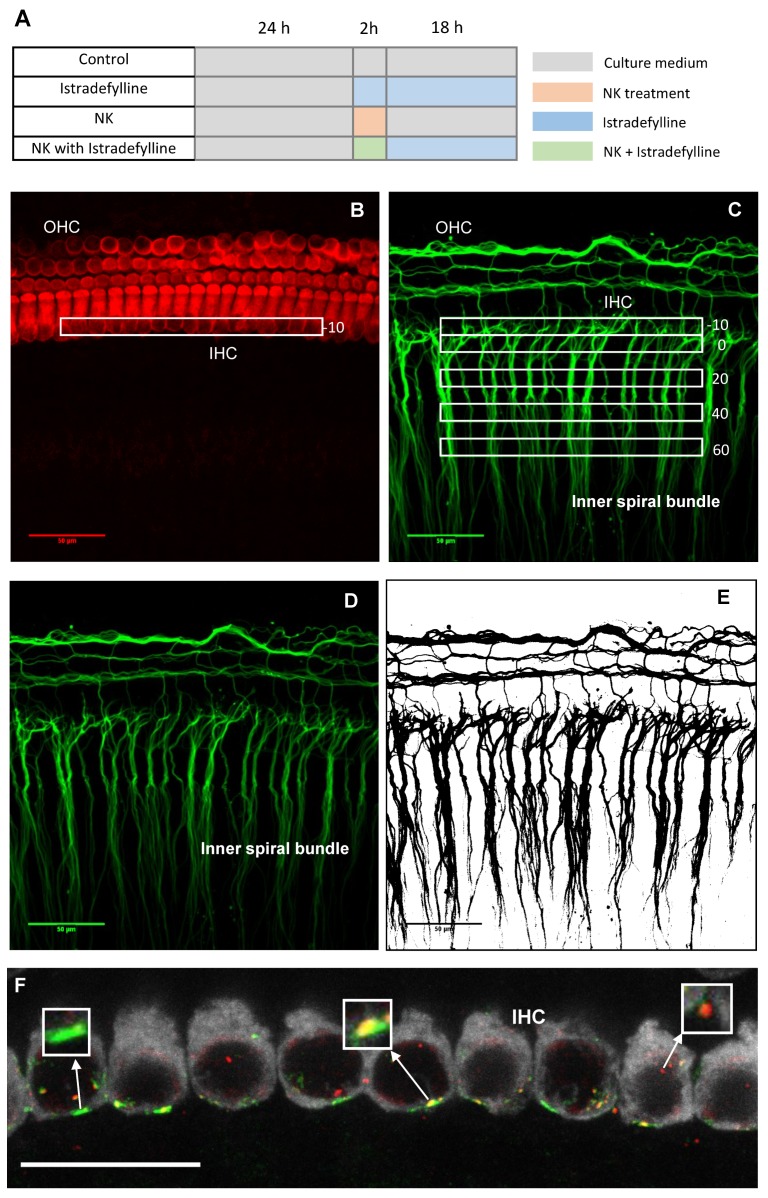
(**A**) Outline of the study. (**B**) The regions of interest (−10 to +60 μm) at the inner hair cell level (myosin VIIa immunolabelling) and (**C**) at the inner spiral bundle level (β-tubulin immunolabelling) in organotypic tissue cultures of the P6 Wistar rat cochlea. (**D**) An original projected image of neurites immunolabelled with β-tubulin and (**E**) the converted binary image used for data analysis. (**F**) An image of the inner hair cell synaptic region: a paired synapse (yellow), orphan postsynaptic density protein (PSD95, green) and orphan presynaptic ribbon (CtBP2, red). Scale bars (**B**–**E**), 50 μm; (**F**) 20 μm.

To model excitotoxic injury, cochlear explants were treated for 2 h with a combination of glutamate receptor agonists NMDA and kainic acid (“NK treatment”; 0.5 mM each in culture medium; In Vitro Technologies). These glutamate receptor agonists were added after pre-incubation of cochlear tissues in normal culture medium for 24 h, necessary for stabilising cochlear tissue following dissection. After 2 h of NK treatment, NK solution was replaced and incubated for another 18 h in normal culture medium (Figure 1A). This time point (18 h) was selected after initial titration of the optimal post-incubation period after NK treatment (6–24 h). To test the effect of A_2A_R inhibition on cochlear tissues, cochlear explants were treated with istradefylline (200 nM; Sigma Aldrich, St. Louis, MO, USA) in 0.1% Dimethyl sulfoxide (DMSO; Sigma Aldrich, St. Louis, MO, USA) for 20 h after the initial 24 h of incubation in normal culture medium (Figure 1A). The effect of istradefylline on NK-induced exitotoxic injury was tested by exposing the cochlear explant to NK in conjunction with istradefylline for 2 h, followed by post-incubation in istradefylline-containing culture medium for 18 h (NK + istradefylline). Cochlear explants were randomly assigned into one of the following groups: control (incubated in normal culture medium; n = 7), NK treatment (n = 7), istradefylline treatment (n = 5), NK with istradefylline (n = 10). Subsequently, the explants were fixed with 4% paraformaldehyde (PFA) in 0.1M phosphate buffer (PB) for 20 min at room temperature (RT).

### 2.4. Immunohistochemistry

The study was restricted to the middle turn of the cochlea to minimise any turn-related differences in the cochlear response to excitotoxic injury. Cochlear explants were immunolabelled with class III β-tubulin which labels spiral ganglion neurons (SGN) and their neurites, myosin VIIa (labels sensory hair cells), PSD95 (labels postsynaptic density protein) and CtBP2 (labels presynaptic ribbons in the inner hair cells). Briefly, after tissue fixation in 4% PFA, the cochlear explants were washed with 0.1 M PBS (3 × 10 min), blocked and permeabilised for 2 h at RT using 10% normal goat serum (NGS) and 2.5% Triton X-100 in 0.1 M PBS. The tissues were labelled using primary antibodies diluted in 0.1 M PBS with 5% NGS and 0.1% Triton X-100 and incubated overnight at 4 °C. The following primary antibodies were used: βΙΙΙ-tubulin IgG2a (1:1000; Abcam, Cambridge, UK), PSD95 mouse IgG2a (1:100; Abcam, Cambridge, UK), CtBP2 mouse IgG1 (1:500; BD Biosciences, Dublin, Ireland) and Myosin VIIa rabbit polyclonal antibody (1:500; Sapphire Biosciences, Redfern, Australia). The next day, cochlear tissues were washed with 0.1 M PBS (3 × 10 min) and incubated for 2 h at RT with the following secondary antibodies: goat anti-mouse IgG2a Alexa-488 (1:500; Invitrogen, Carlsbad, CA, USA), goat anti-mouse IgG1 Alexa-647 (1:500; Invitrogen, Carlsbad, CA, USA) and goat anti-rabbit IgG Alexa-568 (1:500; Invitrogen, Carlsbad, CA, USA). The tissues were then washed with 0.1 M PBS (3 × 10 min) and mounted onto a glass slide using Citifluor AFI mounting medium and stored at 4 °C.

### 2.5. Imaging

Cochlear tissues were imaged using a laser scanning confocal microscope (Olympus FV1000 Live cell system) to visualise the hair cells and their innervation. A z-stack was obtained (60× objective, 1.35 NA) from the myosin and β-tubulin labelled tissues and captured in 1.0 μm steps to quantify the density of SGN neurites connected with the inner hair cells (IHC). Similarly, tissues labelled with myosin VIIa, PSD95 and CtBP2 were imaged using confocal microscopy to assess the density of ribbon synapses in the cochlea. The IHC synapses were imaged at 60× magnification to obtain z stacks in 0.2 μm steps and zoomed (2.4× digital zoom) into 10 IHC. Synaptic ribbons paired with PSD95 were then quantified using a compressed z-stack of each image using image processing software ImageJ. The total number of ribbons was divided by the number of IHC to infer the number of paired synapses per IHC. The images obtained from the β-tubulin and myosin VIIa-labelled tissues were processed using FIJI/Image J (version 1.52 g). A z projection was created for β-tubulin and myosin VIIa channels using the maximum signal intensity of individual slices from each stack. The coordinates of the IHC nucleus were determined using the myosin VIIa channel and a rectangular box (10 μm × 150 μm) was created over the IHC nuclei (Figure 1B). This box with the upper edge positioned 10 μm above the IHC base represents the first region of interest (ROI) and was labelled “−10”. Similarly, a series of rectangular boxes were created in the β-tubulin channel (Figure 1C). These rectangular boxes are various ROI labelled “0”, “20”, “40” and “60” representing a consistent distance (μm) below the IHC base. To analyse the density of the neurites in each ROI, β-tubulin staining was converted into binary format and the β-tubulin projection was expressed as percentage area fraction. The threshold value for binary conversion was determined using the average of the mean and minimum error method in ImageJ. The area fraction in each ROI was recorded and compared across different culture conditions.

The presence of IHC-auditory nerve synapses was determined by identifying the co-localisation of CtBP2 and PSD95 immunofluorescence (Figure 1F). To avoid bias, all images were given random numbers prior to data analysis and the researcher was blinded to experimental conditions.

### 2.6. Adenosine A_2A_R Distribution

The cochleae were extracted from the temporal bones at P6, perfused with 4% PFA through the round window and fixed in 4% PFA overnight at 4 °C. On the following day, the cochleae were washed with 0.1M PBS and decalcified in Shandon TBD-1 Decalcifier (Thermo Fisher) for 30 min. Following wash in PBS, the cochleae were cryoprotected overnight with 30% sucrose in 0.1 M PBS at 4 °C. The cochleae were then mounted in Tissue-Plus Optimal Cutting Temperature Compound (OCT, Fisher Scientific, Hampton, NH, USA), snap-frozen with *n*-pentane and stored at −80 °C. Cochleae were cryosectioned at 25 μm and immunolabelled with A_2A_R-specific antibody using a floating section technique.

Cochlear cryosections were permeabilised with 1% Triton X-100 in 0.1 M PBS and blocked using 10% normal donkey serum for 1 h at RT, followed by incubation with the primary A_2A_R antibody (rabbit anti-mouse, Alomone Labs, Jerusalem, Israel, 1:50 dilution) overnight at 4 °C. In control tissues, the primary antibody was omitted or pre-absorbed for 3 h with the blocking peptide (1:1, Alomone Labs, Jerusalem, Israel). The next day, the cryosections were washed (3 × 10 min) with 0.1 M PBS and incubated with the secondary antibody (Alexa-594 donkey anti-rabbit, 1:400; Life Technologies, Carlsbad, CA, USA) for 2 h at RT. Cochlear tissues were also immunolabelled with Myosin VIIa antibody (1:500; Sapphire Biosciences, Redgerm, Australia) to visualize sensory hair cells and ΒIII-tubulin (1:1000, Abcam, Cambridge, UK) to label SGN neurites. Cochlear tissues were then mounted in Citifluor for imaging. The A_2A_R, myosin VIIa and β-tubulin immunofluorescence was imaged simultaneously under a laser scanning confocal microscope (FluoView™ FV1000, Olympus, Tokyo, Japan) and processed with Olympus FluoView ver.1.7 software. The images were obtained from four animals (eight cochleae) followed by qualitative image analysis.

### 2.7. Statistical Analysis

Results are presented as the mean ± standard error of mean (SEM), and the α level was set at *p* = 0.05. Data were confirmed to have a normal distribution using the Shapiro-Wilk test prior to statistical analysis. Statistical analysis was performed using a one-way ANOVA followed by Sidak’s multiple comparison test GraphPad Prism (v.7 software, San Diego, CA, USA).

## 3. Results

### 3.1. The Effect of NMDA/Kainic Acid (NK) Treatment on Cochlear Explants

**Figure 2:**
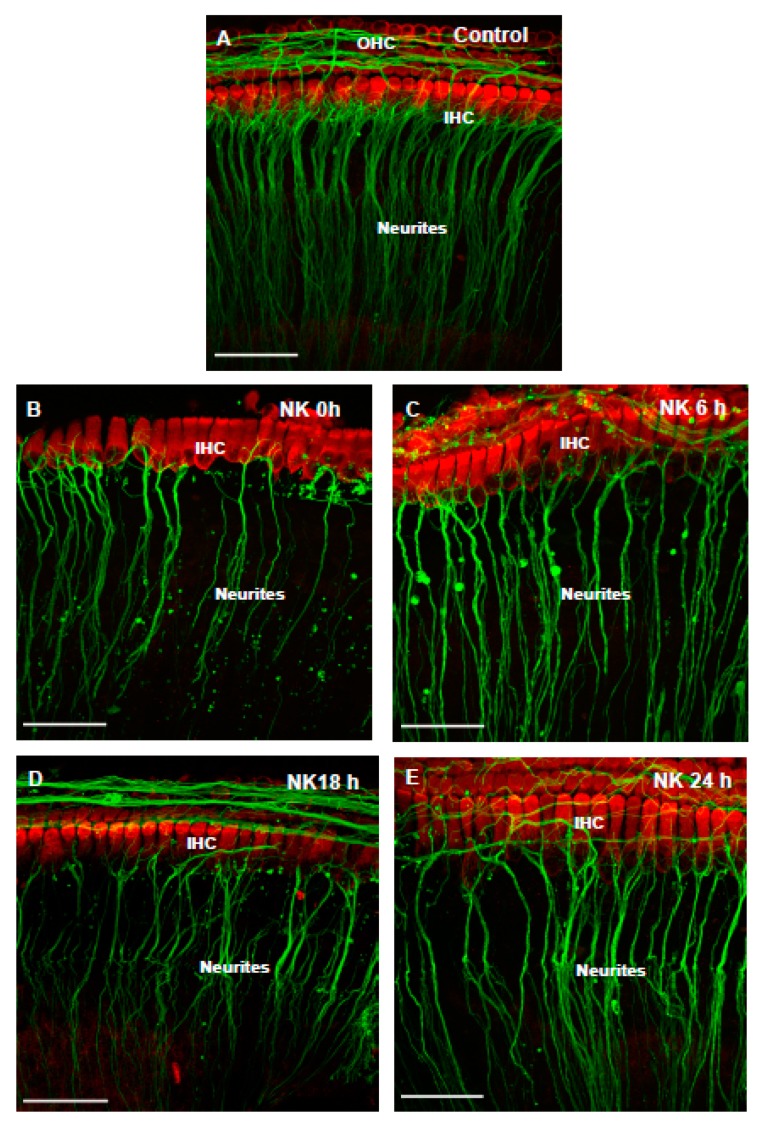
The effect of excitoxic injury in the neonatal rat cochlea induced by co-administration of *N*-methyl-d-aspartate (NMDA) and kainic acid (NK treatment). Images B–E show the NK effect in the function of time in order to find the best post-NK time point for further experiments in the study. (**A**) P6 Wistar rat cochlea labelled with Myosin VIIa (red; sensory hair cells) and β-tubulin (green; neurites of the spiral ganglion neurons) in normal culture medium; (**B**) Immediately after incubation with NK; (**C**) 6 h; (**D**) 18 h; (**E**) 24 h after NK treatment. Images are representative of five experiments per time point. Scale bars, 50 μm.

The structure of the cochlea was well preserved in the normal culture medium, as demonstrated by intact rows of sensory hair cells and abundance of neural processes projected to the hair cells (Figure 2A). NK administration caused extensive loss of the SGN neurites (Figure 2B–E). A retraction and loss of SGN processes innervating the sensory hair cells was evident immediately after the cessation of NK treatment (Figure 2B) and appeared to be severe after 18 h (Figure 2D), hence this time point was selected for further studies. There was evidence of cell debris around the remaining neurites that immunolabelled with β-tubulin (Figure 2B–E). The signs of re-innervation of the hair cells were observed 24 h after NK treatment (Figure 2E) as reported previously [24].

### 3.2. The Effect of Istradefylline on NK-Induced Loss of SGN Neurites

**Figure 3:**
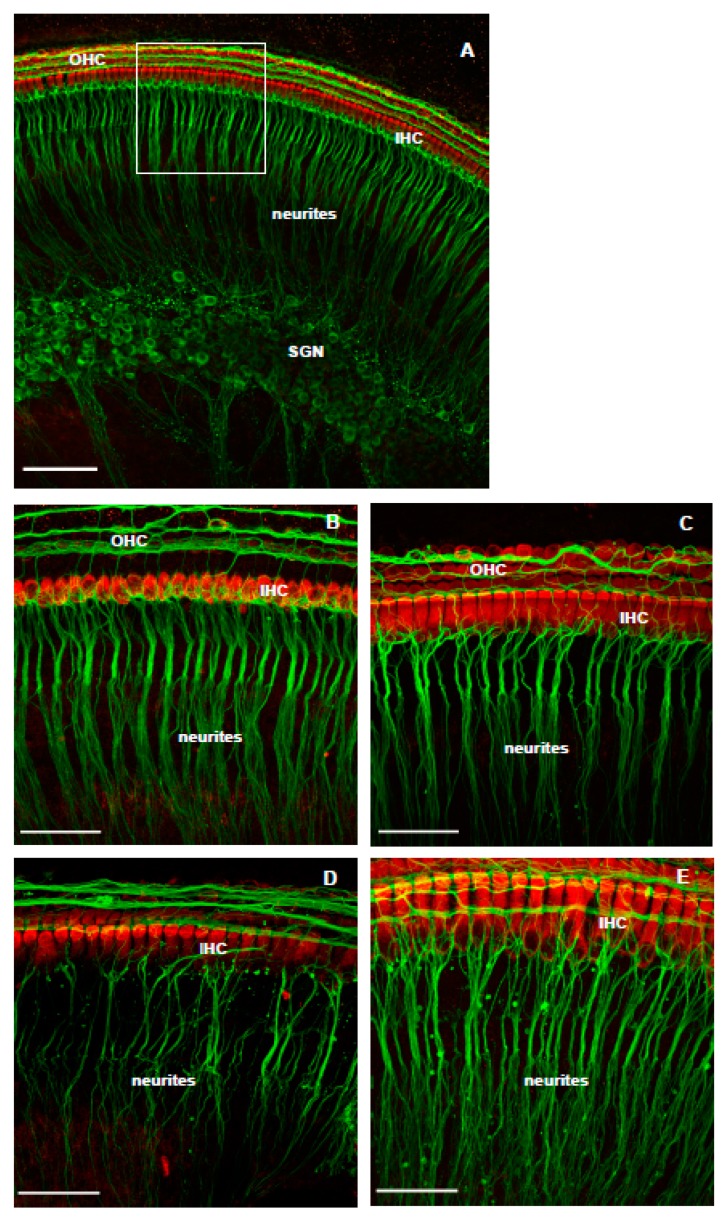
The effect of istradefylline treatment on cochlear explants exposed to excitotoxic injury. (**A**) P6 Wistar rat cochleae labelled with Myosin VIIa (red; sensory hair cells) and β-tubulin (green; spiral ganglion neurons and their neurites) in normal culture medium at low magnification (20×). The white box indicates the region of interest. (**B**) Cochlea cultured in normal medium (n = 7) and (**C**) culture medium containing istradefylline (n = 5). (**D**) Rat cochlea 18 h after NK-induced injury (n = 7) and (**E**) with istradefylline treatment (NK + Istradefylline; n = 10). IHC, inner hair cells; OHC, outer hair cells; SGN, spiral ganglion neurons. Images are representative of the number of experiments shown in brackets for each group. Scale bars, (**A**) 100 μm; (**B**–**E**) 50 μm.

As expected, the cochlear explants in the normal culture medium showed fully preserved sensory hair cells and spiral ganglion neurons (Figure 3A,B). Cochlear explants were also well preserved after incubation with the A_2A_R antagonist istradefylline alone, as demonstrated by an orderly arrangement of the inner and outer hair cells and SGN neurites (Figure 3C). The hair cells were still well preserved 18 h after NK treatment, but the density of SGN neurites was substantially reduced (Figure 3D). Addition of istradefylline to the culture medium significantly improved the density of SGN projections to the IHC (Figure 3E).

**Figure 4:**
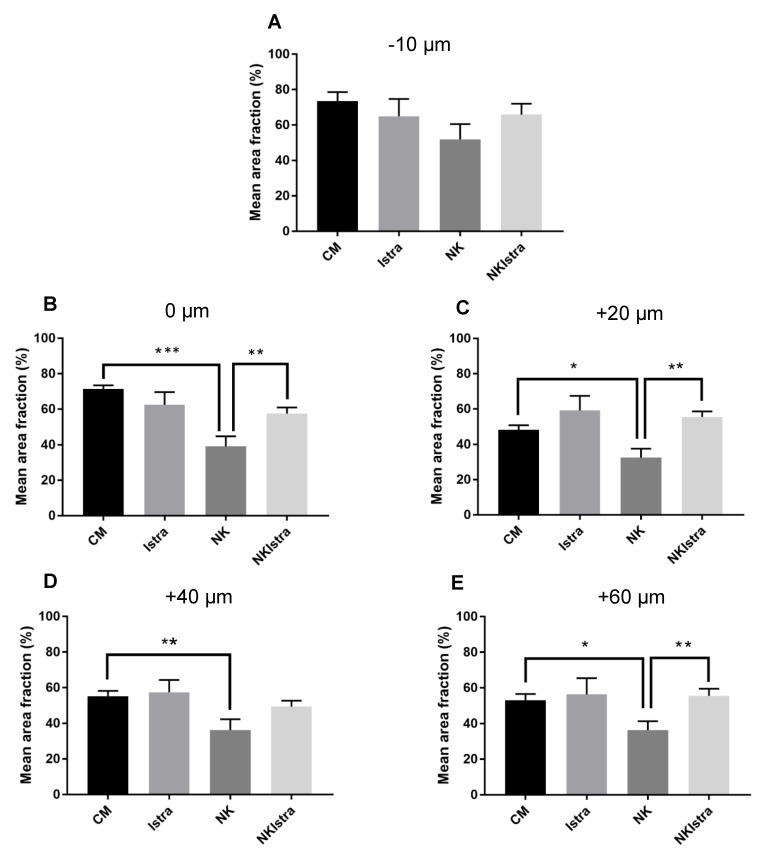
The relative density of SGN neurites in cochlear explants at different regions of interest designated as (**A**) −10 μm, (**B**) 0 μm, (**C**) +20 μm, (**D**) +40 μm and (**E**) +60 μm. CM, normal culture medium (n = 7); Istra, istradefylline (n = 5); NMDA/kainic acid, NK (n = 7), NKIstra, NK + istradefylline (n = 10). Data presented as mean ± SEM. * *p* < 0.05; ** *p* < 0.01; *** *p* < 0.001, one-way ANOVA followed by a post-hoc Sidak’s multiple comparisons test.

The density of SGN neurites in each experimental condition was semi-quantitatively assessed by comparing the area fraction of β-tubulin labelled tissues after conversion to the binary image (Figure 4). The density of neurites was greatest at the level of IHC nucleus and basal regions (−10 μm, 0 μm) in all experimental conditions (Figure 4A,B). Supplementation of istradefylline (200 nM) to the culture medium did not affect the density of neural fibres in any ROI (Figure 4). In contrast, NK administration significantly (*p* = 0.0001 at 0 μm; *p* = 0.0152 at 20 μm; *p* = 0.0091 at 40 μm; *p* = 0.0239 at 60 μm) reduced the density of neural fibres in each ROI below the IHC level (−10). However, administration of istradefylline significantly (*p* = 0.0047 at 0 μm; *p* = 0.0003 at 20 μm; *p* = 0.0063 at 60 μm) improved the density of neural projections in NK-treated tissues to the level similar to the control group incubated in normal culture medium (Figure 4).

### 3.3. The Effect of Istradefylline on Ribbon Synapse Counts After Excitotoxic Injury

**Figure 5:**
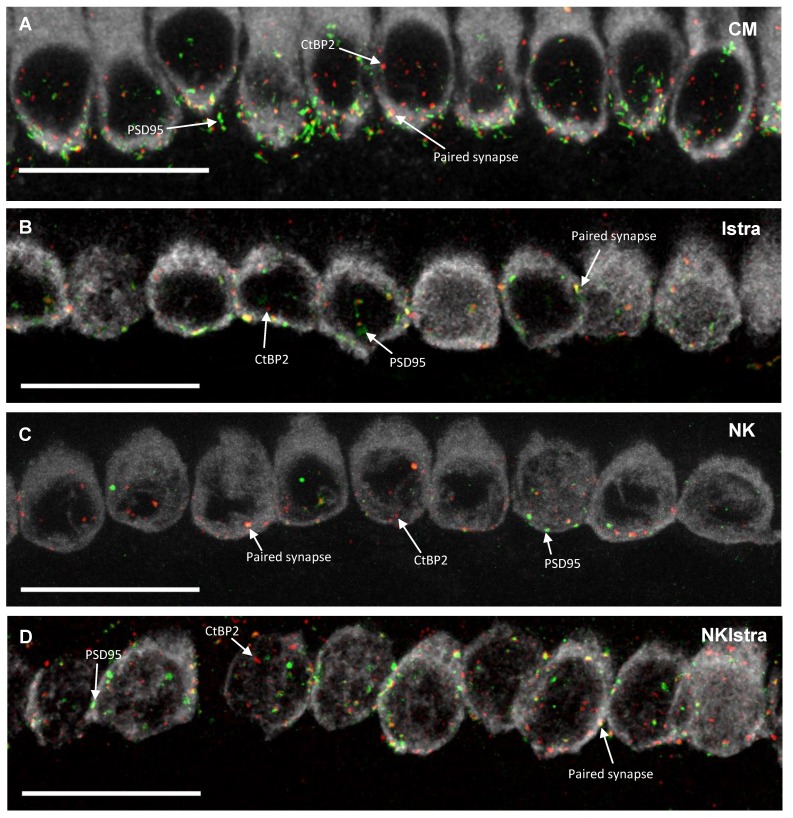

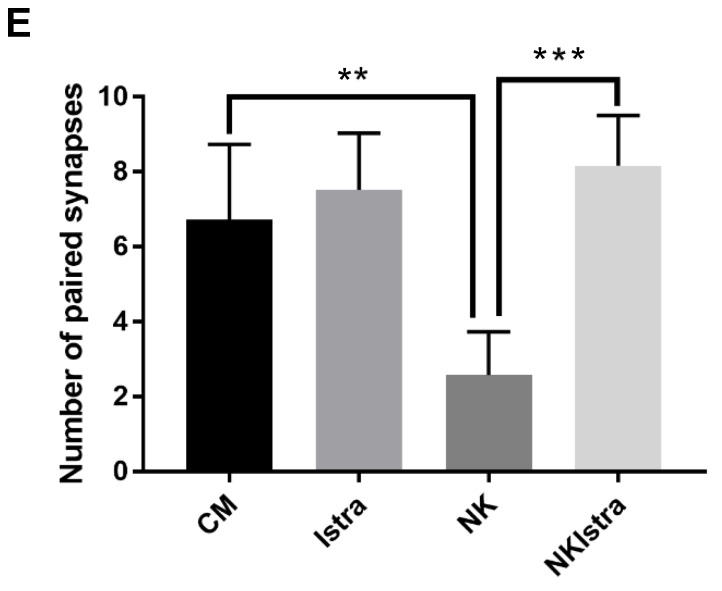
Ribbon synapses in the mid-cochlear region of P6 Wistar rats. Cochlear tissues immunolabelled with PSD95 (postsynaptic density protein, green), CtBP2 (synaptic ribbons, red) and myosin VIIa (inner hair cells, grey) antibodies in (**A**) normal culture medium (CM); (**B**) with addition of istradefylline (Istra); (**C**) with NMDA/kainic acid (NK); (**D**) a combined NKIstra treatment. Scale bars, 20 μm. (**E**) Number of paired synapses per inner hair cell in different treatment groups. Data presented as mean ± SEM. CM, n = 5; Istra, n = 5; NK, n = 4, NKIstra, n = 6. ** *p* < 0.01; *** *p* < 0.001, one-way ANOVA followed by a post-hoc Sidak’s multiple comparisons test. Images A–D are representative of the number of experiments shown for each group.

To identify and quantify afferent synapses, whole mounts of the organ of Corti were immunolabelled with antibodies to CtBP2 (component of the presynaptic ribbon), PSD95 (postsynaptic density protein) and myosin VIIa (sensory hair cells). A paired synapse was identified as the one with co-localisation of CtBP2 and PSD95 positive puncta, while an orphan synapse was the one with presynaptic or postsynaptic elements only (Figure 5). The number of paired synapses in the developing rat cochlea was counted using the ImageJ cell counter tool. In the present study, 6.7 ± 2.0 paired synapses per IHC were counted in the mid-cochlear region (40–60% distance from the apex) in the control tissues incubated in normal culture medium (Figure 5E). The number of paired synapses was similar in the control and istradefylline-treated groups (Figure 5E). NK administration reduced the numbers of paired synapses to 2.5 ± 1.2 per IHC (*p* = 0.0032 vs. control), however the addition of istradefylline reversed the effect of NK and restored the number of ribbon synapses (*p* = 0.0001 vs. NK; Figure 5E).

### 3.4. Distribution of Adenosine A_2A_R in Afferent Synapses of the Developing Rat Cochlea

**Figure 6:**
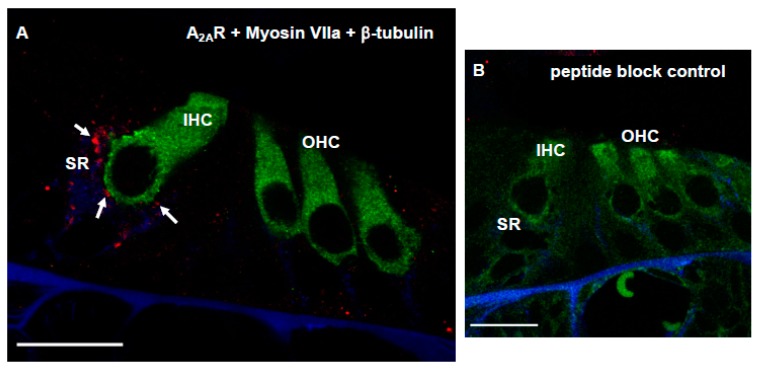
Confocal images of the P6 Wistar rat cochlea showing adenosine A_2A_ receptor (A_2A_R) distribution at the synaptic region (SR) of the inner hair cells (arrows). (**A**) A_2a_R immunofluorescence (red), Myosin VIIa immunolabelling of the sensory hair cells (green) and β-tubulin labelling of SGN neurites (blue). (**B**) Peptide block control. Images are representative of eight cochleae obtained from four animals. Scale bars, 20 μm.

The distribution of A_2A_R in the synaptic region of the developing (P6) rat cochlea was investigated using cochlear cryosections of the sensory epithelium. Cross-sections of the developing cochlea were immunolabelled with the adenosine A_2A_R antibody (Alomone Labs) and imaged using a laser scanning confocal microscope. In the organ of Corti, A_2A_R was immunolocalised in the synaptic region (SR) of the IHC (red dots, Figure 6A). A_2A_R immunolabelling was predominantly observed in the postsynaptic region of the IHC (co-localisation with β-tubulin), whilst presynaptic localisation (co-localisation with Myosin VIIa) was negligible (Figure 6A). Immunofluorescence was absent in cochlear sections after antigen adsorption with the blocking peptide (Figure 6B).

## 4. Discussion

In this study we investigated the effect of adenosine A_2A_R inhibition on cochlear rescue from excitotoxic injury using organotypic tissue cultures of the developing (P6) rat cochlea. NK administration caused the loss of afferent synapses and the retraction of SGN neurites in cochlear tissues, reaching the peak at 18 h after NK administration. Supplementation of the culture medium with istradefylline, a clinically approved A_2A_R antagonist, substantially reduced NK excitotoxicity and rescued SGN neurites. Istradefylline on its own did not have any negative effect on the viability of sensorineural tissues in the cultured cochlea. The findings from this study support a strong neuroprotective role of istradefylline in a rat model of excitotoxic cochlear injury. Immunolocalisation of the A_2A_R in the synaptic region of the IHC is consistent with the predominantly postsynaptic effect of istradefylline.

Neonatal cochlear explants have been used in auditory research to manipulate cell responses and investigate signalling pathways using pharmacological agents [25]. An advantage of this technique is that it can be used to monitor changes during development and disease pathogenesis and screen for candidate therapeutic compounds [26]. In comparison with homogenous mammalian cell lines, cochlear explants can better model the cochlear microenvironment [26], however they still cannot fully replicate complex cellular interactions in live animals [25]. In this study we have used the cochlea from P6 Wistar rats to reduce the variability caused by the ongoing cochlear development. The P6 cochlea is more mature and begins to resemble the adult cochlea, making it more suitable for quantitative assessment of neural injury and recovery from injury [24].

### 4.1. The Effect of NK-Induced Injury and Treatment with Istradefylline on SGN Neurites

Neurotransmission at the IHC-SGN synapse includes both NMDA and non-NMDA (kainate and AMPA-type) glutamate receptors [27,28,29]. In the developing rat cochlea, AMPA and NMDA receptors in nerve endings of afferent fibres are associated with the sensory hair cells from the first postnatal day onwards [30]. NMDA receptors expressed in SGN dendritic terminals play a critical role in IHC-SGN neurotransmission before the onset of hearing [31]. Both NMDA and kainate receptors are overactivated following NK administration causing excitotoxic injury in the developing cochlea. NK administration can induce a cascade of intracellular events similar to that evoked by excessive glutamate release from the IHC. Glutamate receptors are predominantly expressed postsynaptically, but they may also have a presynaptic localisation at the IHC [29,32]. Activation of postsynaptic glutamate receptors results in membrane depolarisation and rapid ion influx, leading to Ca^2+^ overload and accumulation of reactive oxygen species [33]. These intracellular changes ultimately lead to mitochondrial dysfunction and activation of caspases, followed by neuronal apoptosis [34]. Kainate receptor-mediated excitotoxicity also results in apoptotic cell death as it triggers the release of lysosomal enzymes [35]. In organotypic tissue cultures, NK treatment causes rapid degeneration of type 1 SGN neurites, but not acute death of SGN [24]. However, glutamate receptor agonists do not disrupt type 2 SGN-outer hair cell synapses in vitro [24] or in vivo [36].

In the current study, NK treatment was used to mimic glutamate excitotoxicity, which is a well-established mechanism of HHL and noise-induced hearing loss. The loss of afferent synapses in the presence of intact hair cells resembles cochlear neuropathy induced by noise exposure [2]. Noise exposure has additional presynaptic effects in the sensory hair cells, hence the NK excitotoxicity model allowed investigation of the postsynaptic effects whilst avoiding damage to the hair cells. NK treatment induced deafferentation of sensory hair cells similar to previous studies [24]. The effect of NK-induced injury and treatment with istradefylline was assessed using immunofluorescence and semi-quantitative analysis of SGN neurite density. The current study shows that the hair cell morphology and the density of SGN neurites are similar in tissues incubated in normal culture medium and culture medium supplemented with istradefylline, suggesting that the application of istradefylline does not affect the cellular make-up of cochlear explants. The most salient point of this study is that the administration of istradefylline improves the density of SGN neurites and the number of afferent synapses after NK treatment and thus promotes cochlear rescue from excitotoxic injury. Postsynaptic localisation of the A_2A_R points at the most likely target of istradefylline in the synaptic region.

### 4.2. The Effect of NK-Induced Injury and Istradefylline Treatment on Ribbon Synapses

The effect of NK excitotoxicity was also assessed by measuring the density of paired ribbon synapses (co-localisation of CtBP2 and PSD95-positive puncta) in the cochlear epithelium. The number of paired synapses observed in the NK-treated tissues was substantially lower than in the control group, which can be attributed to decoupling of sensory hair cells from their innervation. The number of paired synapses in tissues treated with istradefylline was similar to that observed in tissues incubated in the normal culture medium, suggesting that administration of istradefylline does not affect synaptic connections in the cochlea. However, administration of istradefylline significantly improved the density of ribbon synapses after NK treatment, consistent with increased density of SGN neurites.

These findings are consistent with our previous studies showing excellent preservation of cochlear ribbon synapses in adult A_2A_R null mice after traumatic noise exposure [20]. However, careful consideration is required to interpret the functional significance of paired ribbon synapses in the developing rat cochlea before the onset of hearing [37]. Whilst istradefylline improves the density of SGN neurites and synaptic ribbons in the developing cochlea, the neuroprotective effect in live adult animals requires further investigation.

Both A_1_R and A_2A_R are known to modulate synaptic transmission and influence neuronal sensitivity to neurotransmitters [11,38]. Postsynaptic A_1_R increase the threshold to open NMDA receptor channels and thus lower the chance of postsynaptic membrane depolarization, whilst A_2A_R operates in an opposing manner to regulate neuronal excitability [38]. A_2A_R is widely distributed in brain synapses, where it plays an important role in synaptic plasticity, facilitating glutamate release and potentiating NMDA receptor-mediated neurotransmission [39]. Inhibition of A_2A_R is a well-established mechanism of neuromodulation and neuroprotection [40]. A_2A_R inhibition mitigates early-onset cognitive deficits after traumatic brain injury in mice by reducing the phosphorylation of tau proteins in the dentate gyrus [41]. Antagonists of A_2A_R can reverse memory impairments, both in aging rodents [42] and in animal models of Alzheimer’s disease [43]. In addition, istradefylline has been used clinically for treatment of l-DOPA-resistant patients with Parkinson’s disease to reduce cognitive and motor side-effects [44]. Collectively, these studies indicate therapeutic potential of A_2A_R antagonists in CNS disorders. The current study demonstrates that A_2A_R are strategically positioned to modulate postsynaptic responses at the IHC-SGN synapse. Inhibition of the A_2A_R may dampen the postsynaptic glutamate receptor sensitivity to excessive ligands, or alter the transport of ions to reduce activation of postsynaptic neurons [11]. As A_2A_R stimulation activates mitogen-activated protein (MAP) kinases p38, ERK1/2 and JNK1/2 [39], receptor inhibition may also prevent apoptotic events induced by A_2A_R-mediated activation of MAP kinases in SGN.

## 5. Conclusions

NK excitotoxicity in organotypic tissue cultures of the developing rat cochlea provides an appropriate injury model for cochlear neuropathy as the excessive stimulation of the postsynaptic glutamate receptors causes IHC deafferentation without loss of sensory hair cells. The current study investigated the role of A_2A_R inhibition in cochlear rescue from excitotoxic injury. Addition of istradefylline improved the survival of afferent synapses and contributed to the maintenance of the SGN neurite population. These findings suggest a neuroprotective role of istradefylline in a rat model of excitotoxic cochlear injury, with possible implications for the treatment of cochlear neuropathy.
